# Is There a Link between Different Types of Alcoholic Drinks and Obesity? An Analysis of 280,183 UK Biobank Participants

**DOI:** 10.3390/ijerph17145178

**Published:** 2020-07-17

**Authors:** Elif Inan-Eroglu, Lauren Powell, Mark Hamer, Gary O’Donovan, Mitch J. Duncan, Emmanuel Stamatakis

**Affiliations:** 1 The Boden Collaboration for Obesity, Nutrition, Exercise & Eating Disorders, Faculty of Medicine and Health, The University of Sydney, Sydney NSW 2050, Australia; elif.inaneroglu@sydney.edu.au; 2 Prevention Research Collaboration, School of Public Health, Faculty of Medicine and Health, The University of Sydney, Sydney NSW 2050, Australia; lauren.powell@sydney.edu.au; 3 Faculty of Medical Sciences, University College London, London WC1E 6BT, UK; m.hamer@ucl.ac.uk; 4 Facultad de Medicina, Universidad de los Andes, Bogotá 57, Colombia; g.odonovan@uniandes.edu.co; 5 School of Medicine & Public Health; Faculty of Health and Medicine, The University of Newcastle, University Drive, Callaghan NSW 2308, Australia; mitch.duncan@newcastle.edu.au; 6 Priority Research Centre for Physical Activity and Nutrition, The University of Newcastle, University Drive, Callaghan NSW 2308, Australia; 7 Charles Perkins Centre Epidemiology Unit, The University of Sydney, Sydney NSW 2050, Australia

**Keywords:** obesity, adiposity, alcohol, alcoholic drinks, adults

## Abstract

Understanding the associations between types of alcoholic drinks and adiposity has public health relevance, considering that adult overweight and obesity prevalence are increasing worldwide. We aimed to evaluate the association between overall alcohol consumption and types of alcohol drinks with markers of adiposity from the UK Biobank baseline data (*n* = 280,183, 48.3% female). Generalized linear models were used to examine the associations between alcohol consumption with body mass index (BMI) and body fat percentage. Those drinking within the public health guidelines had a lower BMI by 1.34 kg/m^2^ (95% CI 1.42, 1.26 kg/m^2^) compared to never drinkers. Association between alcohol consumption and body fat percentage were not statistically significant. Compared to those who never drink wines (red wine, champagne and fortified wine), drinkers of these alcoholic beverages had lower BMI (difference of −0.75 kg/m^2^, 95% CI −0.78, −0.72 kg/m^2^; −0.48 kg/m^2^, 95% CI −0.52, −0.45 kg/m^2^; and −0.24 kg/m^2^, 95% CI −0.29, −0.18 kg/m^2^, respectively). Beer and spirits drinkers had higher BMI compared to never drinkers of beer and spirits (difference of 0.18 kg/m^2^, 95% CI 0.14, 0.22 kg/m^2^ and 0.64 kg/m^2^, 95% CI 0.61, 0.68 kg/m^2^, respectively). Our data did not find a link between alcohol drinking and higher risk of obesity.

## 1. Introduction

Obesity is a key risk factor for cardiovascular disease, type 2 diabetes and some cancers that has reached epidemic proportions, particularly in high-income countries [1]. In 2015, 62% of adults in the UK were considered overweight (body mass index (BMI) > 25 kg/m^2^) or obese (BMI > 30 kg/m^2^) [2]. The World Health Organization (WHO) predicts that by 2030 36% of men and 33% of women in the UK will be obese [3]. Obesity develops when energy intake chronically exceeds energy expenditure resulting in an energy imbalance and resultant weight gain [1]. Therefore, dietary habits are a key determinant of body weight status [4].

Alcohol is a unique component of the human diet which has a relatively high energy content of 7.1 kcal/g while it stimulates appetite and limits satiety development [5]. Consequently, alcohol intake may be a key contributor to the development of overweight and obesity. Epidemiological investigations of the association between alcohol intake and BMI have produced inconsistent results [6,7,8,9,10,11]. The definition of alcohol consumption differs across previous studies that examined the association between alcohol and obesity. For instance, some studies defined alcohol consumption as quantity or frequency alone [12,13]. The inclusion of previous drinkers to never drinkers group might also contribute to contradictory findings since previous drinkers might stop drinking due to health issues and might have higher or lower weight than never drinkers [13,14]. Additionally, the current inconsistent evidence might be explained by the small sample size and a relatively small number of heavy drinkers in these cross-sectional studies [8]. In addition, adiposity markers differ across studies. Several studies used weight as an adiposity marker, whereas others used BMI or waist circumference [7,8]. Another possible explanation for these conflicting findings is that different types of alcoholic drinks may have differential associations on body weight [15,16,17]. For instance, the consumption of spirits and beer, but not of wine, has been associated with increased body weight [15]. Other recent studies showed no association or an inverse association between wine consumption and weight gain [18,19,20]. Overall, there is a paucity of evidence on the associations of specific alcoholic drinks consumption and markers of adiposity.

Understanding the associations between types of alcoholic drinks and adiposity has public health relevance, considering that adult overweight and obesity prevalence is increasing worldwide [21], that alcohol consumption in the UK and many other countries is very high [22] and that both obesity and alcohol consumption are related to many chronic diseases [23,24,25]. The objective of this study was to examine the associations of overall alcohol consumption and types of alcoholic drinks with adiposity in a large sample of adults residing in England.

## 2. Materials and Methods

This research has been conducted using the UK Biobank Resource under Application Number 25813. The UK Biobank is a large, population-based prospective cohort study. Around 9.2 million invitations were mailed to recruit 502,616 adults (response rate 5.5%) aged 40–69 years between 2006 and 2010 from 22 centers across the UK to reflect a broad socioeconomic demographic and mixture of urban and rural residents. In this study, we excluded participants with missing data in alcohol consumption (*n* = 117,499), physical activity (PA) (calculated as Metabolic Equivalent Task (MET)-hours of PA/week) (*n* = 73,059), body fat percentage (*n* = 7763) or other covariates included in the analysis (*n* = 12,682). We also excluded participants with a BMI <18.5 kg/m^2^ (*n* = 8968) or implausible values of sedentary behavior (>23 h/day) (*n* = 2462). Detailed study methods have been published elsewhere [26]. All participants provided informed consent, and ethical approval was provided by the National Health Service, National Research Ethics Service (Ref 11/NW/0382).

### 2.1. Outcomes

We calculated BMI from the participant’s weight (kg) and height (m^2^) which were measured by trained staff. A BMI ≥ 25 kg/m^2^ was considered overweight, and ≥30 kg/m^2^ was considered obese. Participants with a BMI < 18.5 kg/m^2^ were excluded from all analyses because of the high likelihood of underlying disease, which may have affected both weight status and alcohol drinking behavior. Body fat (BF) percentage was measured by bioimpedance using the Tanita BC-418MA device (Tanita, Tokyo, Japan) using procedures documented in detail elsewhere [27]. The validity of bioelectrical impedance analysis has been previously shown [28].

### 2.2. Alcohol Consumption

The touch-screen self-administered questionnaire was used to collect the data. Participants were asked to classify their current alcohol drinking status as never, previous or current. Current drinkers were asked additional questions regarding their average weekly consumption of alcoholic drink types, such as “In an average week, how many glasses of red wine would you drink?” We calculated the level of overall alcohol consumption as the number of UK units of alcohol consumed per week; the sum of average weekly intake of red wine; champagne and white wine; beer and cider; spirits; fortified wine; and other alcoholic drinks. In the UK, one unit of alcohol is equal to 10 mL of absolute alcohol equivalent [29]. In line with previous research [30], participants were grouped into five categories in terms of total alcohol intake: (1) never drinker; (2) previous drinkers; (3) within guidelines (<14 UK units of alcohol/week for women and <21 units/week for men); (4) hazardous (14–34 units/week in women and 21–48 units/week in men); and (5) harmful (>35 units/week in women and >49 units/week in men). Intake of alcoholic drink types was classified based on participants’ self-reported consumption of each drink type during the previous week. We present the alcohol consumption categorization of previous papers as Supplementary Materials (Table S1).

### 2.3. Covariates

We included age, sex, diet, socioeconomic status, sleep (hours/night), major illness, PA [31], smoking status and sedentary behavior in the model as covariates. PA was quantified using the short-form International Physical Activity Questionnaire (IPAQ) [31]. MET-hours of PA/week was calculated by multiplying the MET value of activity by the number of hours/week. PA was then classified as inactive (≤7.5 MET-hours/week), active at the lower PA guideline (>7.5 MET-hours/week) or active at the upper PA guideline range (>15 MET-hours/week). We adjusted for chronic illness using a dichotomous variable denoting the presence/absence of major cardiovascular disease (ICD-10 codes I01.0 to I199) or cancer (C00.0 to D48.9). Fruit and vegetable consumption (servings/day) was used as a marker of dietary quality. Participants were asked to report the number of servings of cooked vegetables, salad and raw vegetables, fresh fruit and dried fruit they consumed each day. For example, “On average, how many heaped tablespoons of salad or raw vegetables would you eat per day?” One piece of fruit, such as a banana, or one heaped tablespoon of vegetables was considered as one serving. Sleep was quantified based on participants’ responses to the question “About how many hours sleep do you get in every 24 h? (including naps)” Sedentary time was calculated by summing the total time spent watching television, using a computer screen or driving. Participants with implausible values of >23 h/day were excluded from the analysis. We used the Townsend deprivation index as an indicator of socioeconomic status, which assigns each participant a score relative to the output area (the smallest UK census area) in which their postcode was located, with higher scores indicate greater socioeconomic deprivation [32].

### 2.4. Statistical Analysis

We calculated the Pearson correlation coefficients to determine the correlation between BMI and body fat percentage. We used generalized linear models to examine the associations between overall alcohol consumption and consumption of alcoholic drink types with BMI and body fat percentage. The first model was adjusted for age and sex only. The second model included the additional covariates of smoking status, sleep duration, sedentary behavior, chronic illness, PA, Townsend deprivation index and daily fruit and vegetable consumption. We did not use education in the main analyses due to the large many missing data which would considerably compromise the analytic sample size. We also adjusted the models that investigated alcoholic drink types (consuming alcohol type vs. not consuming alcohol type (referent)) and adiposity markers for all Model 2 covariates and overall alcohol consumption. To determine the dose–response of alcoholic drink types with BMI, we ran fully-adjusted generalized linear models for each alcoholic drink type grouped as follows: those who did not report consumption of the alcoholic drink type in the week prior to measurement; low consumption defined as less than or equal to the median for the alcoholic drink type; and high consumption defined as greater than the median for the alcoholic drink type. We also performed sex-stratified generalized linear models to determine dose–response of alcoholic drink types with BMI by adjusting for age, overall alcohol consumption, smoking status, sleep (h/night), sedentary behavior (h/day), illness (major cardiovascular disease or cancer), physical activity, Townsend deprivation index and daily fruit and vegetable consumption. We performed additional sex-stratified generalized linear models for each alcoholic drink type. We carried out a set of sensitivity analyses:

(a) Multiple logistic regression to investigate the associations between overall alcohol consumption and alcoholic drink types with dichotomous (BMI-defined) overweight (25 kg/m^2^) and obesity (30 kg/m^2^).
(b) Additional adjustment for education as an individual-level indicator of socioeconomic status using age completed the highest education qualification.
(c) Additional analysis by including 2389 participants who were underweight but had a BMI over 13 kg/m^2^.
(d) We combined previous and never drinkers as a reference in order to compare the results with previous studies.

All analyses were conducted using SPSS Version 22.0 (IBM, Chicago, IL, USA).

## 3. Results

Table 1 shows participants’ baseline characteristics according to alcohol consumption category. The mean BMI ranged 26.7–28.1 kg/m^2^ and the mean BF% ranged 31.4–31.6% across alcohol consumption categories. The mean age ranged 55.6–56.8 years. About 8.8% of the sample reported not drinking any alcohol currently. Sleep duration was similar across alcohol consumption categories (ranged 7.1–7.2 h). About 82.5% of never drinkers also never smoked, and 11.9% of never drinkers were previous smokers. Sedentary behavior duration was the least for within guideline drinkers (4.6 h/day). Fruit and vegetable consumption was highest for never drinkers (9.0 average number of servings/day), whereas it was lowest for harmful drinkers (7.0 average number of servings/day).

The correlation between BMI and body fat percentage was −0.003 (*p* = 0.18).

The mean age of individuals across different alcohol types showed similar results (ranged 52.9–58.6 years). Consumption of different alcohol types was similar for females and males except for beer. About 21% of females indicated that they were beer drinkers vs. 79% of males.

Our formal interaction tests showed a statistically significant alcohol*sex interaction for total alcohol intake (*p* < 0.01), red wine (*p* < 0.01), champagne/white wine (*p* < 0.01), beer (*p* < 0.01), spirits (*p* < 0.01) and fortified wine (*p* < 0.01) in the multivariable analysis examining the association between BMI and both overall and different alcoholic drink types, whereas there was not significant alcohol*sex interaction for other alcohol (*p* = 0.997). We did not find statistically significant alcohol*sex interaction for total alcohol intake (*p* = 0.337), red wine (*p* = 0.274), champagne/white wine (*p* = 0.250), beer (*p* = 0.729), spirits (*p* = 0.417), fortified wine (*p* = 0.820) and other alcohol (*p* = 0.165) in the multivariable analysis examining the association between body fat percentage and both overall and different alcoholic drink types.

### 3.1. Overall Alcohol Consumption, BMI and Body Fat

Table 2 shows the associations between BMI and body fat percentage with alcohol consumption. In the minimally adjusted model (Model 1), we found statistically significant associations between BMI and alcohol consumption categories, whereas we did not find an association with body fat percentage. Individuals who consumed alcohol within guidelines and at hazardous levels had lower BMI compared with never drinkers (GLM coefficient −1.34 kg/m^2^, 95% CI −1.42 to −1.26 kg/m^2^ and GLM coefficient −0.85 kg/m^2^, 95% CI −0.93 to −0.77 kg/m^2^, respectively). In the fully adjusted model (Model 2), the differences for consumption within guideline levels and hazardous drinkers (GLM coefficient −1.12 kg/m^2^, 95% CI −1.20 to −1.05 kg/m^2^; and GLM coefficient −0.71 kg/m^2^, 95% CI −0.79 to −0.63 kg/m^2^, respectively) persisted, although they were attenuated. Harmful level drinkers also had lower BMI compared with never drinkers in Model 1 (GLM coefficient −0.09 kg/m^2^, 95% CI −0.24 to 0.07 kg/m^2^). This association was higher in Model 2 (GLM coefficient −0.28 kg/m^2^, 95% CI −0.43 to −0.13 kg/m^2^). The sensitivity analysis with the inclusion of underweight participants (BMI > 13 kg/m^2^) produced similar results with the main analysis (Table S2). For instance, within guideline drinkers had lower BMI than never drinkers in the minimally adjusted model (GLM coefficient −1.22 kg/m^2^, 95% CI −1.29 to −1.14 kg/m^2^). As in the main analysis, these associations remain the same, although it was slightly attenuated. In addition, there was not statistically significant association between overall consumption and body fat percentage. The sensitivity analyses with education adjustment produced comparable results pointing towards slight attenuation (e.g., GLM coefficient −1.02 kg/m^2^, 95% CI −1.12 to −0.93 kg/m^2^ for within guideline drinkers and GLM coefficient −0.66 kg/m^2^, 95% CI −0.77 to −0.56 kg/m^2^ for hazardous drinkers). There were no statistically significant associations between alcohol consumption and body fat percentage (Table S3). The results of the multiple logistic regression with obesity and overweight (including obesity) as outcomes (Table S4) produced results that were in agreement with the main analyses. For example, drinkers within guidelines displayed lower odds of overweight (including obesity) compared with never drinkers (odds ratio 0.70, 95% CI 0.67 to 0.73). In addition, the odds of obesity were lower in within guideline drinkers and hazardous drinkers (odds ratio 0.59, 95% CI 0.57–0.61 and odds ratio 0.70, 95% CI 0.67–0.73, respectively) compared with never drinkers. Furthermore, additional sensitivity analyses by combining previous and never drinkers showed that there were statistically significant associations between BMI and alcohol consumption categories in parallel with the main analysis (Table S5). For instance, within guideline drinkers had lower BMI than never/previous drinkers (GLM coefficient −1.33, 95% CI −1.39 to −1.28) in Model 1. This association was not seen in Model 2.

### 3.2. Individual Types of Alcohol Drinks, BMI and Body Fat

Table 3 presents the adjusted associations between BMI and body fat percentage with individual types of alcoholic drink consumption by sex. In the minimally adjusted model, we found a statistically significant association between BMI and individual types of alcoholic drink, whereas we did not find any association with body fat percentage. Red wine, champagne/white wine and fortified wine drinkers had lower BMI compared to never drinkers of each type of alcoholic drink (GLM coefficient −0.75 kg/m^2^, 95% CI −0.78 to −0.72 kg/m^2^, GLM coefficient −0.48 kg/m^2^, 95% CI −0.52 to −0.45 kg/m^2^ and GLM coefficient −0.24 kg/m^2^, 95% CI −0.29 to −0.18 kg/m^2^, respectively). Beer and spirits drinkers had higher BMI compared to never drinkers of each type of alcoholic drink (GLM coefficient 0.18 kg/m^2^, 95% CI 0.14–0.22 kg/m^2^ and GLM coefficient 0.64 kg/m^2^, 95% CI 0.61–0.68 kg/m^2^, respectively). Associations for beer and BMI were not attenuated (GLM coefficient 0.20 kg/m^2^, 95% CI 0.16–0.24 kg/m^2^) following adjustment for potential confounders. Associations were slightly attenuated but still statistically significant for red wine, champagne/white wine, spirits and fortified wine (GLM coefficient −0.53 kg/m^2^, 95% CI −0.57 to −0.50 kg/m^2^, GLM coefficient −0.38 kg/m^2^, 95% CI −0.41 to −0.35 kg/m^2^ and GLM coefficient −0.18 kg/m^2^, 95% CI −0.23 to −0.12 kg/m^2^, respectively). The associations between BMI with red wine, champagne/white wine and fortified wine of both female and male drinkers were similar to the main analysis. In addition, female spirits and other alcohol drinkers had higher BMI compared to never drinkers of each type of alcoholic drink (GLM coefficient 0.71 kg/m^2^, 95% CI −0.65 to 0.76 kg/m^2^ and GLM coefficient 0.73 kg/m^2^, 95% CI 0.37–1.09 kg/m^2^, respectively) whereas male beer/cider and spirits drinkers had higher BMI compared to never drinkers of each type of alcoholic drink (GLM coefficient 0.32 kg/m^2^, 95% CI 0.27–0.37 kg/m^2^ and GLM coefficient 0.57 kg/m^2^, 95% CI 0.53–0.62 kg/m^2^, respectively). The sensitivity analysis with the inclusion of underweight participants (BMI > 13 kg/m^2^) produced results that were in line with the main analysis (Table S6). For example, we found that red wine, champagne/white wine and fortified wine drinkers had a lower BMI than never drinkers of each type of alcoholic drinks (GLM coefficient −0.52 kg/m^2^, 95% CI −0.55 to −0.48 kg/m^2^, GLM coefficient −0.37 kg/m^2^, 95% CI −0.41 to −0.34 kg/m^2^ and GLM coefficient −0.18 kg/m^2^, 95% CI −0.24 to −0.13 kg/m^2^, respectively), whereas beer/cider, spirits and other alcohol drinkers had a higher BMI than never drinkers of each type of alcoholic drinks drink (GLM coefficient 0.21 kg/m^2^, 95% CI 0.17–0.25 kg/m^2^, GLM coefficient 0.56 kg/m^2^, 95% CI 0.52–0.59 kg/m^2^ and GLM coefficient 0.30 kg/m^2^, 95% CI 0.06–0.55 kg/m^2^, respectively). Table S7 shows that the associations between types of alcoholic drink and BMI were similar to the main analysis. For instance, red wine, champagne/white wine and fortified wine drinkers were less likely to be overweight (including obese) compared with those who did not report consumption of each alcoholic drink type (odds ratio 0.87, 95% CI 0.86–0.89; odds ratio 0.91, 95% CI 0.89–0.92 and odds ratio 0.94, 95% CI 0.91–0.96, respectively). Beer and spirits drinkers had higher odds of being overweight (including obese) than those who did not report consumption of each alcoholic drink type (odds ratio 1.17, 95% CI 1.14–1.19 and odds ratio 1.28, 95% CI 1.26–1.30, respectively).

### 3.3. Dose–Response Associations of Specific Alcoholic Drink Types and BMI/obesity

**Figure 1:**
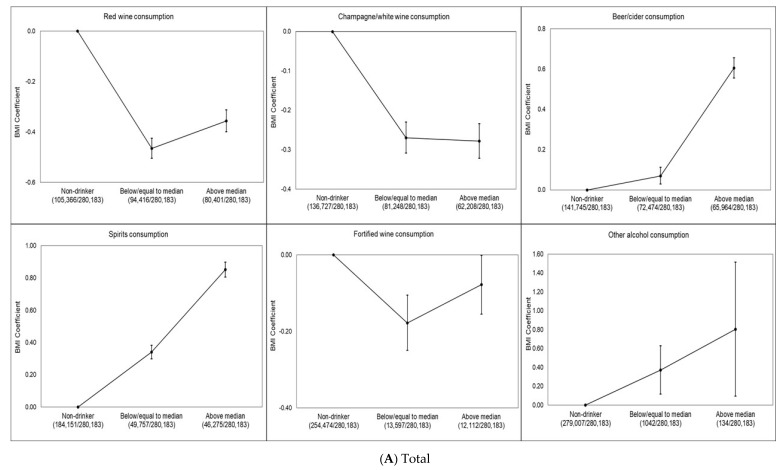

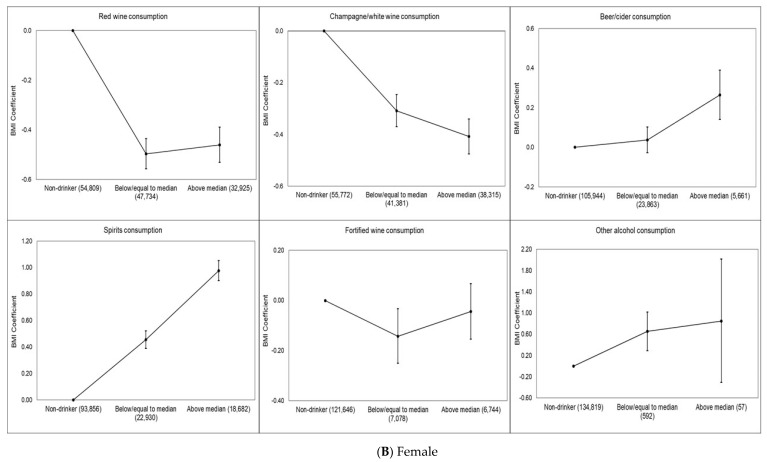

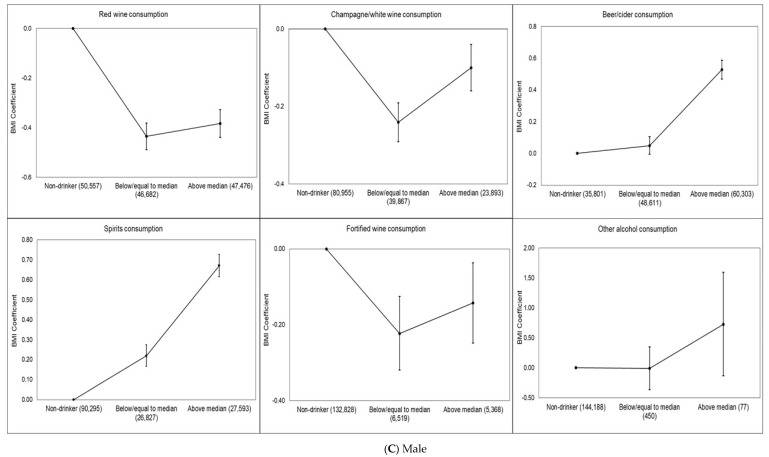
(**A**–**C**) Multivariable-adjusted dose–response association of alcoholic beverage types and BMI in the UK Biobank. The BMI coefficient displays the mean difference between the reference category (no consumption in last week) and the other consumption categories. The model is adjusted for age, sex, overall alcohol consumption, smoking status, sleep (h/night), sedentary behavior (h/day), illness (major cardiovascular disease or cancer), physical activity, Townsend deprivation index and daily fruit and vegetable consumption for the total sample. The model is adjusted for age, overall alcohol consumption, smoking status, sleep (h/night), sedentary behavior (h/day), illness (major cardiovascular disease or cancer), physical activity, Townsend deprivation index and daily fruit and vegetable consumption for each sex. Individuals who did not report consumption of the alcoholic beverage type in the week prior to measurement were considered non-drinkers. Low consumption was defined as less than or equal to the median for the alcoholic beverage type, and high consumption was defined as greater than the median for the alcoholic beverage type. The number of participants in each alcohol consumption category (i.e., non-drinker, low or high) included in the analysis is shown below each figure.

Dose–response associations between BMI and each type of drink for total sample and by sex are shown in Figure 1A–C. Red wine (−0.46 kg/m^2^, 95% CI −0.50 to −0.42 kg/m^2^), champagne/white wine (−0.27 kg/m^2^, 95% CI −0.31 to −0.23 kg/m^2^) and fortified wine (−0.18 kg/m^2^, 95% CI −0.25 to −0.10 kg/m^2^) drinkers (defined as low consumption) had significantly lower BMI than never drinkers of this alcohol type. These associations were slightly attenuated for red wine (−0.36 kg/m^2^, 95% CI −0.40 to −0.31 kg/m^2^) and fortified wine (−0.08 kg/m^2^, 95% CI −0.15 to −0.002 kg/m^2^) drinkers (defined as high consumption), whereas slightly increased for champagne/white wine (−0.28 kg/m^2^, 95% CI −0.32 to −0.23 kg/m^2^) drinkers (defined as high consumption) compared to never champagne/white wine drinkers. In contrast, beer (0.07 kg/m^2^, 95% CI 0.03–0.11 kg/m^2^) and spirit (0.34 kg/m^2^, 95% CI 0.30–0.11 kg/m^2^) drinkers (defined as low consumption) had higher BMI than never drinkers (Figure 1A). We found that female red wine (−0.50 kg/m^2^, 95% CI −0.56 to −0.44 kg/m^2^), champagne/white wine (−0.31 kg/m^2^, 95% CI −0.37 to −0.25 kg/m^2^) and fortified wine (−0.14 kg/m^2^, 95% CI −0.25 to −0.03 kg/m^2^) drinkers (defined as low consumption) had significantly lower BMI than never drinkers of this alcohol type. These associations were slightly attenuated for red wine (−0.46 kg/m^2^, 95% CI −0.53 to −0.39 kg/m^2^) and fortified wine (−0.04 kg/m^2^, 95% CI −0.15 to 0.07 kg/m^2^) drinkers (defined as high consumption) whereas slightly increased for champagne/white wine (−0.41 kg/m^2^, 95% CI −0.48 to −0.34 kg/m^2^) drinkers (defined as high consumption) compared to never champagne/white wine drinkers as it was in the total population. We did not find any dose–response association for female beer drinkers. Female spirit (0.45 kg/m^2^, 95% CI 0.39–0.52 kg/m^2^) and other alcohol (0.65 kg/m^2^, 95% CI 0.29–1.02 kg/m^2^) drinkers (defined as low consumption) had higher BMI than never drinkers and this association slightly increased for female high consumption of spirit (0.98 kg/m^2^, 95% CI 0.90–1.05 kg/m^2^) and other alcohol (0.85 kg/m^2^, 95% CI −0.31to 2.02 kg/m^2^) and drinkers (Figure 1B). Male red wine (−0.43 kg/m^2^, 95% CI −0.49 to −0.38 kg/m^2^) and champagne/white wine (−0.24 kg/m^2^, 95% CI −0.29 to −0.19 kg/m^2^) drinkers (defined as low consumption) had significantly lower BMI than never drinkers of these alcohol types. These associations were slightly attenuated for red wine (−0.38 kg/m^2^, 95% CI −0.44 to −0.32 kg/m^2^) and champagne/white wine (−0.10 kg/m^2^, 95% CI −0.16 to −0.04 kg/m^2^) drinkers (defined as high consumption). Male fortified wine (−0.14 kg/m^2^, 95% CI −0.25 to −0.04 kg/m^2^) drinkers (defined as high consumption) had lower BMI compared to never fortified wine drinkers. In contrast, male beer (0.53 kg/m^2^, 95% CI 0.47 to 0.59 kg/m^2^) and spirit (0.67 kg/m^2^, 95% CI 0.61 to 0.73 kg/m^2^) drinkers (defined as high consumption) had higher BMI than never drinkers (Figure 1C).

## 4. Discussion

Our study is among the first to examine the association between different type of alcoholic drinks with key adiposity markers. We found a statistically significant association between overall alcohol consumption status and BMI, but not with body fat percentage. Drinkers within guidelines had lower BMI than never drinkers and previous drinkers. The sensitivity analyses of with obesity and overweight (including obesity) as outcomes produced similar results with the main analyses, which highlights the robustness of our data. While previous studies showed a direct association between alcohol consumption and BMI [6,7,8], other studies have shown inverse or no associations [10,11]. Several hypotheses have been proposed to explain the possible inverse association between alcohol and weight. Firstly, in addition to its acute effects, alcohol has a physiological impact on appetite and heart rate, which in turn may contribute to the total energy balance [5]. Alcohol increases the insulin sensitivity of skeletal muscles which can reduce body fat storage by reducing lipogenesis [33]. There is growing evidence that alcohol consumption may stimulate caloric loss through a microsomal ethanol-oxidizing system [34,35]. In addition, alcohol-based calories might substitute dietary calories among moderate drinkers, without increasing total energy intake [16]. Besides biological explanations, we cannot rule out the possibility of residual confounding whereby the seemingly “protective” associations we observed were due to residual (unmeasured) socioeconomic differences and poorly measured confounders such as overall diet. In the UK, higher socioeconomic groups tend to be exposed to better health-related lifestyles than lower socioeconomic groups [36,37].

The UK Chief Medical Officers’ low-risk drinking guidelines advise that not to drink more than 14 units (175 mL wine equals 2 units) a week regularly for men and women to keep health risks from alcohol low [30]. About 24% of adults in England and Scotland regularly drink over the guidelines and 27% of drinkers in the UK binge drink on their heaviest drinking days (over eight units for men and over six units for women) [38,39,40] and therefore exceed the alcohol consumption recommendation of the Mediterranean diet, which includes a moderate intake of red wine (one glass a day for women and two glasses a day for men) [41]. The inverse association between overall alcohol consumption status and BMI must be interpreted with caution. This result might be interpreted as moderate alcohol consumption being a part of a healthy diet. However, any alcohol consumption is related to increased risk of premature mortality and many chronic diseases, including cancer, type 2 diabetes and cardiovascular diseases [22,23,30,42].

High consumption of beer and spirits, but not red wine, champagne/white wine and fortified wine, was associated with a higher risk of overweight/obesity but not with body fat percentage. These differential results by adiposity markers may be explained by the different properties of BMI and body fat percentage. A meta-analysis of 25 studies showed that commonly used BMI cut-off values to diagnose obesity have high specificity, but low sensitivity to identify adiposity, as they fail to identify half of the people with excess body fat percentage [43]. Although BMI is the most frequently used index to assess obesity and is usually used as a proxy for body fatness in large epidemiological studies because of its simplicity, it has been criticized due to its effectiveness on differentiating between muscle and fat tissue and therefore reflecting of true body fatness [44,45].

Previous cohort studies also found similar findings regarding the association between the consumption of different types of alcoholic drinks and BMI [18,46,47]. In a previous study of Sayon-Orea et al., beer and spirits consumption (≥7 drinks/week) was associated with a 119 g/year higher average yearly weight gain, whereas no association was found between wine consumption and overweight/obesity [18]. Two large-scale American cohorts, namely the Nurses’ Health Study (*n* = 121,700) and Health Professionals Follow Up Study (*n* = 51,529) [46], also found positive associations between BMI and the consumption of spirits. In addition to having a higher BMI, a direct association was found between waist circumference and high consumption of beer and spirits, while the inverse association was found between wine consumption and waist circumference in the prospective Copenhagen City Heart Study [47]. In a cross-sectional English study (*n* = 8864), beer and spirit consumption significantly increased the odds of obesity relative to wine consumption [48]. In contrast to these studies, irrespective of the type of drink, positive associations between alcohol consumption and general adiposity (BMI and body fat percentage) and to a greater extent, central adiposity (waist to hip ratio and waist circumference) has also been shown [8].

A possible explanation for these results is that the type of alcohol drink preference may be associated with different health-related behaviors, such as beer and spirit drinkers have poorer diets and lifestyle habits than wine drinkers [15,49]. For instance, Ruidavets et al. [49] showed that wine drinkers had higher physical activity levels than beer or mixed drinkers. In the same study, wine drinking was associated with a smaller percentage of current smokers and wine drinkers had higher consumption of vegetables and fruit and the diet quality index compared to beer drinkers. In a study of Mortensen et al. [50], wine drinking was significantly associated with higher parenteral education level as well as higher socioeconomic status than beer drinking. The authors concluded that wine drinking is a general indicator of optimal social, cognitive and personality development. In addition, wine consumption has been associated with a lower BMI [48]. Red wine can increase aromatase expression, which can elevate the local estradiol concentrations and consequently leads to a lower weight gain [51]. Beulens et al. showed that moderate consumption of red wine for four weeks leads to an increased level of adiponectin which is involved in fatty acid oxidation [52]. Overall, the discrepancies between studies could potentially be attributed mainly to methodological differences including inclusion and exclusion criteria, assessment of alcohol intake and obesity measures, categorizing alcohol intake and subject characteristics. In addition, since obesity is a multi-factorial condition affected by numerous factors including lifestyle habits, environment and genetics, determining the independent effect of alcohol consumption on weight is difficult [1,15].

We found that the association between different types of alcoholic drinks and BMI varied between females and males, indicating that female spirits and other alcohol drinkers and male beer/cider and spirit drinkers had higher BMI compared to never drinkers of each type of alcoholic drink. This situation can be explained by the socio-cultural norms about alcohol and the differences by sex in alcohol metabolism that reflect differences in body fat between males and females [15,53]. Another explanation might be that alcohol drinkers might not compensate for the energy intake from alcohol by consuming less energy from food. This is particularly obvious for the spirits and beer drinkers compared to wine drinkers [48,54].

The primary strength of this study is the large sample size. We were also able to use two objective obesity markers. However, there are also several limitations. The cross-sectional study design does not allow us to infer anything about causality. Residual confounding may explain some of the statistically significant findings. We also did not have any data related to the weight history of participants which might partially explain some of the statistically significant results. Lastly, alcohol intake was self-reported and underreporting [11] is possible. Only around 5% of the target population took part in UK Biobank, and study members typically showed more favorable risk profiles than the non-responders, which is a possible source of bias. Nevertheless, select groups of the population tend to demonstrate similar risk factor–disease associations to those seen in the general population [55].

## 5. Conclusions

In the present study, we found that BMI was inversely related to overall alcohol consumption. There was no association between body fat percentage and overall alcohol consumption. We also showed that red wine, champagne/white wine and fortified wine drinkers had a lower BMI compared to never drinkers of each type of alcoholic drink, whereas beer and spirits drinkers had higher BMI compared to never drinkers of each type of alcoholic drink. Within the limitations of its cross-sectional design, our study did not find a link between overall alcohol consumption and higher risk of obesity. Further longitudinal studies will allow us to better understand the causal associations between alcohol and risk of obesity and overweight.

## Figures and Tables

**Table 1:** Characteristics of study sample by level of alcohol consumption (*n* = 280,183).

	Alcohol Consumption Categories				
Characteristic	Never Drinker (*n* = 13,143)	Previous Drinker (*n* = 11,676)	Within Guidelines (*n* = 175,812)	Hazardous (*n* = 75,626)	Harmful (*n* = 3926)
Alcohol consumption (number of UK units/week) ^1^	N/A	N/A	6.8 (3.2)	21.4 (7.3)	57.0 (22.8)
Red wine (percent who reported any consumption in the previous week)	N/A	N/A	65.9	74.4	67.3
Champagne/white wine (percent who reported any consumption in the previous week)	N/A	N/A	55.2	58.2	60.4
Beer/cider (percent who reported any consumption in the previous week)	N/A	N/A	48.5	67.3	60.2
Spirits (percent who reported any consumption in the previous week)	N/A	N/A	32.2	49.0	61.8
Fortified wine (percent who reported any consumption in the previous week)	N/A	N/A	9.7	10.8	11.5
Other alcohol (percent who reported any consumption in the previous week)	N/A	N/A	0.5	0.4	0.7
*Physical activity* ^2^					
Not meeting guidelines (%)	21.2	21.1	16.8	16.8	23.0
Meeting lower guidelines (%)	17.8	16.3	18.1	17.0	17.0
Meeting upper guidelines (%)	60.9	62.5	65.1	66.2	59.9
Age (years)	56.8 (8.6)	56.8 (8.0)	56.2 (8.1)	56.5 (7.9)	55.6 (7.8)
Female (%)	69.3	52.7	54.7	29.7	40.3
Body mass index (BMI, kg/m^2^) ^3^	27.9 (5.4)	28.1 (5.4)	26.7 (4.3)	27.5 (4.1)	28.1 (4.7)
Body mass index (BMI, kg/m^2^) ^3^ categories					
Normal (%)	31.5	30.6	37.3	27.7	25.7
Overweight (%)	39.5	39.4	43.5	48.6	43.0
Obese (%)	29.0	30.0	19.2	23.8	31.3
Body fat percentage (BF%)	31.4 (8.5)	31.5 (8.6)	31.4 (8.5)	31.4 (8.5)	31.6 (8.5)
Sleep duration (h)	7.1 (1.3)	7.2 (1.4)	7.2 (1.0)	7.2 (1.0)	7.2 (1.3)
*Cigarette smoking*					
Never (%)	82.5	45.1	58.5	38.5	24.4
Previous (%)	11.9	40.6	34.3	46.7	44.8
Current (%)	5.5	14.3	7.2	14.8	30.7
Sedentary behavior (hours/day)	4.7 (2.7)	5.1 (2.8)	4.6 (2.3)	5.0 (2.4)	5.4 (2.9)
Townsend deprivation index ^4^	−0.5 (3.4)	−0.2 (3.5)	−1.8 (2.8)	−1.5 (2.9)	−0.6 (3.3)
Fruit and vegetable consumption (average number of servings/day)	9.0 (6.2)	8.7 (5.7)	8.1 (4.4)	7.6 (4.4)	7.0 (5.1)
*Chronic Illness*					
Major CVD event (%)	3.6	3.6	3.6	3.7	3.3
Cancer (%)	8.4	8.3	8.0	8.0	7.5
Education ^5^	16.3 (4.5)	16.3 (3.2)	16.8 (2.8)	16.8 (2.7)	16.7 (2.9)
*Employment Status (%)*					
In paid employment/self-employed	56.9	57.1	57.3	57.4	57.5
Unemployed	1.7	1.5	1.6	1.7	1.8
Retired	33.2	33.9	33.3	33.3	32.7

Data are presented as mean (standard deviation) unless indicated otherwise. ^1^ Alcohol consumption categories are based on the average weekly intake of standard drinks relative to UK guidelines. In the UK, one standard drink equals 10 mL of pure alcohol. Within guidelines: <14 units/week in women and <21 units/week in men; hazardous: 14–35 units/week in women and 21–49 units/week in men; harmful: >35 units/week in women and >49 units/week in men. The alcohol types indicate the percentage of individuals who reported consuming this alcoholic beverage within the last week. ^2^ Physical activity (PA) patterns were classified based on the World Health Organization PA guidelines as not meeting guidelines (<150 min moderate physical activity (MPA)/week), meeting lower PA guideline (150–299 min MPA/week) and meeting upper PA guideline (≥300 min MPA/week). Each minute spent performing vigorous PA counted as two minutes of MPA. ^3^ Body mass index (BMI) = Weight (kg)/height (m^2^). ^4^ Townsend deprivation index scores ranged −6 to 11. Scores were derived from national census data. Each participant was assigned a score relative to the output area in which their postcode was located. Higher scores reflect a higher degree of socioeconomic deprivation. ^5^ Age of completion of full-time education.

**Table 2:** Multivariable-adjusted associations between BMI and body fat percentage with alcohol consumption in the UK Biobank (*n* = 280,183).

	Model 1		Model 2	
	Coefficient (95% CI)	*p*	Coefficient (95%CI)	*p*
Alcohol consumption				
*BMI*				
Never drinker	Referent	<0.001	Referent	<0.001
Previous drinker	−0.01 (−0.12, 0.10)		−0.11 (−0.22, −0.01)	
Within guidelines	−1.34 (−1.42, −1.26)		−1.12 (−1.20, −1.05)	
Hazardous	−0.85 (−0.93, −0.77)		−0.71 (−0.79, −0.63)	
Harmful	−0.09 (−0.24, 0.07)		−0.28 (−0.43, −0.13)	
*Body fat percentage*				
Never drinker	Referent	0.63	Referent	0.55
Previous drinker	0.16 (−0.05, 0.37)		0.16 (−0.05, 0.38)	
Within guidelines	0.06 (−0.09, 0.21)		0.07 (−0.08, 0.23)	
Hazardous	0.06 (−0.10, 0.22)		0.08 (−0.09, 0.24)	
Harmful	0.21 (−0.09, 0.52)		0.23 (−0.08, 0.54)	

Generalized linear model coefficient; mean differences (in risk factor values) between the reference category (never drinker) and each of the other alcohol consumption categories. Model 1 is adjusted for age and sex only. Model 2 is adjusted for age, sex, smoking status, sleep (h/night), sedentary behavior (h/day), illness (major cardiovascular disease or cancer), physical activity, Townsend deprivation index and daily fruit and vegetable consumption. Alcohol consumption categories are based on the average weekly intake of standard drinks relative to UK guidelines. In the UK, one standard drink equals 10 mL of pure alcohol. Within guidelines: <14 units/week in women and <21 units/week in men; hazardous: 14–35 units/week in women and 21–49 units/week in men; harmful: >35 units/week in women and >49 units/week in men. Body mass index (BMI) = Weight (kg)/height (m^2^). A BMI ≥ 25 was considered overweight, and ≥30 was considered obese. Physical activity (PA) was classified based on the Metabolic Equivalent Task (MET) scores of participants’ responses to the International Physical Activity Questionnaire (IPAQ) as inactive (≤7.5 MET-hour/week), active at the lower PA guideline (>7.5 MET-hour/week) or active at the upper PA guideline (>15 MET-hour/week). MET-hours/week were calculated based on the average number of minutes per day spent walking for any purpose, minutes/day in moderate PA and vigorous PA. Townsend deprivation index scores were derived from national census data. Each participant was assigned a score relative to the output area in which their postcode was located. Higher scores reflect a higher degree of socioeconomic deprivation.

**Table 3:** Multivariable-adjusted associations between BMI and body fat percentage with types of alcoholic beverages consumed in the UK Biobank (*n* = 280,183).

	Model 1		Model 2	
	Coefficient (95% CI)	*p*	Coefficient (95% CI)	*p*
Alcohol consumption (ref: not drinking this drink type)				
*Total*				
*BMI*				
Red wine	−0.75 (−0.78, −0.72)	<0.001	−0.53 (−0.57, −0.50)	<0.001
Champagne/white wine	−0.48 (−0.52, −0.45)	<0.001	−0.38 (−0.41, −0.35)	<0.001
Beer/cider	0.18 (0.14, 0.22)	<0.001	0.20 (0.16, 0.24)	<0.001
Spirits	0.64 (0.61, 0.68)	<0.001	0.54 (0.51, 0.58)	<0.001
Fortified wine	−0.24 (−0.29, −0.18)	<0.001	−0.18 (−0.23, −0.12)	<0.001
Other alcohol	0.56 (0.31, 0.81)	<0.001	0.28 (0.04, 0.52)	0.02
*Body fat percentage*				
Red wine	0.03 (−0.04, 0.10)	0.37	0.03 (−0.04, 0.10)	0.42
Champagne/white wine	−0.06 (−0.13, 0.01)	0.08	−0.07(−0.14, 0.001)	0.05
Beer/cider	−0.03 (−0.11, 0.04)	0.41	−0.04 (−0.12, 0.04)	0.29
Spirits	0.02 (−0.05, 0.09)	0.61	0.02 (−0.06, 0.09)	0.67
Fortified wine	0.01 (−0.10, 0.13)	0.82	0.02 (−0.10, 0.13)	0.79
Other alcohol	0.23 (−0.27, 0.72)	0.37	0.23 (−0.27, 0.72)	0.37
*Females*				
*BMI*				
Red wine	−0.88 (−0.93, −0.83)	<0.001	−0.52 (−0.58, −0.47)	<0.001
Champagne/white wine	−0.69 (−0.74, −0.64)	<0.001	−0.41 (−0.47, −0.36)	<0.001
Beer/cider	0.03 (−0.03, 0.09)	0.32	0.06 (0.002, 0.12)	0.04
Spirits	0.71 (0.65, 0.76)	<0.001	0.68 (0.63, 0.73)	<0.001
Fortified wine	−0.24 (−0.33, −0.16)	<0.001	−0.12 (−0.20, −0.04)	0.01
Other alcohol	0.73 (0.37, 1.09)	<0.001	0.49 (0.15, 0.84)	0.01
*Body fat percentage*				
Red wine	0.01 (−0.08, 0.11)	0.78	0.02 (−0.09, 0.12)	0.76
Champagne/white wine	−0.02 (−0.11, 0.08)	0.75	−0.02 (−0.12, 0.09)	0.77
Beer/cider	−0.05 (−0.16, 0.07)	0.41	−0.05 (−0.16, 0.06)	0.39
Spirits	−0.01 (−0.11, 0.09)	0.84	0.003 (−0.10, 0.11)	0.95
Fortified wine	0.04 (−0.12, 0.19)	0.66	0.04 (−0.12, 0.19)	0.65
Other alcohol	−0.07 (−0.73, 0.60)	0.85	−0.04 (−0.70, 0.63)	0.91
*Males*				
*BMI*				
Red wine	−0.64 (−0.68, −0.59)	<0.001	−0.55 (−0.60, −0.51)	<0.001
Champagne/white wine	−0.30 (−0.35, −0.26)	<0.001	−0.28 (−0.32, −0.23)	<0.001
Beer/cider	0.32 (0.27, 0.37)	<0.001	0.19 (0.14, 0.24)	<0.001
Spirits	0.57 (0.53, 0.62)	<0.001	0.40 (0.36, 0.44)	<0.001
Fortified wine	−0.26 (−0.34, −0.19)	<0.001	−0.24 (−0.31, −0.16)	<0.001
Other alcohol	0.32 (−0.02, 0.66)	0.07	0.03 (−0.31, 0.36)	0.88
*Body fat percentage*				
Red wine	0.06 (−0.04, 0.16)	0.25	0.06 (−0.04, 0.16)	0.26
Champagne/white wine	−0.11 (−0.20, −0.02)	0.02	−0.11 (−0.21, −0.02)	0.02
Beer/cider	−0.02 (−0.12, 0.08)	0.71	−0.04 (−0.15, 0.07)	0.47
Spirits	0.04 (−0.05, 0.14)	0.37	0.03 (−0.07, 0.13)	0.53
Fortified wine	−0.01 (−0.17, 0.15)	0.91	−0.01 (−0.17, 0.16)	0.94
Other alcohol	0.59 (−0.15, 1.33)	0.12	0.57 (−0.16, 1.31)	0.13

Generalized linear model coefficient; mean differences (in risk factor values) between participants who did not consume the relevant alcohol type (the referent) and participants who reported consuming the relevant alcohol type. Model 1 is adjusted for age and sex only for the total sample. Model 2 is adjusted for age, sex, overall alcohol consumption, smoking status, sleep (h/night), sedentary behavior (h/day), illness (major cardiovascular disease or cancer), physical activity, Townsend deprivation index and daily fruit and vegetable consumption for the total sample. Model 1 is adjusted for age only for each sex. Model 2 is adjusted for age, overall alcohol consumption, smoking status, sleep (h/night), sedentary behavior (h/day), illness (major cardiovascular disease or cancer), physical activity, Townsend deprivation index and daily fruit and vegetable consumption for each sex. Overall alcohol consumption categories are based on the average weekly intake of standard drinks relative to UK guidelines. In the UK, one standard drink equals 10 mL of pure alcohol. Within guidelines: <14 units/week in women and <21 units/week in men; hazardous: 14–35 units/week in women and 21–49 units/week in men; harmful: >35 units/week in women and >49 units/week in men. Body mass index (BMI) = Weight (kg)/height (m^2^). A BMI ≥ 25 was considered overweight, and ≥30 was considered obese. Physical activity (PA) was classified based on the Metabolic Equivalent Task (MET) scores of participants’ responses to the International Physical Activity Questionnaire (IPAQ) as inactive (≤7.5 MET-hour/week), active at the lower PA guideline (>7.5 MET-hour/week) or active at the upper PA guideline (>15 MET-hour/week). MET-hours/week were calculated based on the average number of minutes per day spent walking for any purpose, minutes/day in moderate PA and vigorous PA. Townsend deprivation index scores were derived from national census data. Each participant was assigned a score relative to the output area in which their postcode was located. Higher scores reflect a higher degree of socioeconomic deprivation.

## Supplementary Materials

The following are available online at https://www.mdpi.com/1660-4601/17/14/5178/s1, Table S1: Alcohol consumption categorization of previously published papers related to alcohol consumption and obesity, Table S2: Multivariable-adjusted associations between alcohol consumption and BMI (including underweight participants) and body fat percentage in the UK Biobank according to UK Drinking Guidelines categorization (*n* = 282,572), Table S3: Multivariable-adjusted associations between BMI and body fat percentage with alcohol consumption in the UK Biobank with adjustment for education (*n* = 171,772), Table S4: Multiple logistic regression describing the associations between alcohol consumption and overweight including obesity in the UK Biobank (*n* = 281,588), Table S5: Multivariable-adjusted associations between BMI and body fat percentage with alcohol consumption in the UK Biobank with combined never/previous drinkers (*n* = 280,183), Table S6: Multivariable-adjusted associations between types of alcoholic beverages consumed and BMI (including underweight participants) and body fat percentage in the UK Biobank (*n* = 282,572), Table S7: Multiple logistic regression describing the associations between overweight and obesity with types of alcoholic beverages in the UK Biobank (*n* = 281,588).
