# Supplementary material for: Is There a Link between Different Types of Alcoholic Drinks and Obesity? An Analysis of 280,183 UK Biobank Participants
Source: Int J Environ Res Public Health. 2020 Jul 17;17(14):5178. doi: 10.3390/ijerph17145178 (PMC7400254; doi:10.3390/ijerph17145178)
Supplement: Supplementary file 1 [file ijerph-17-05178-s001.pdf]

Table S1. Alcohol consumption categorization of previously published papers related to alcohol consumption and obesity.

Reference	Alcohol Consumption Categorization
Baek, S. I., So, W. Y. Relationship between obesity in Korean adolescents and the frequency of alcohol consumption, the amount of alcohol consumed, and the frequency of severe alcohol intoxication. *Obesity research & clinical practice* **2012**, *6*, e159–e166.	Never<br>1—2 times per month<br>3—5 times per month<br>6—9 times per month<br>10—19 times per month<br>20—30 times per month
Wannamethee, S. G.; Shaper, A. G. Alcohol, body weight, and weight gain in middle-aged men. The American journal of clinical nutrition **2003**, *77*, 1312–1317.	None-occasional<br>Light-moderate<br>Heavy
Lahti-Koski, M., Pietinen, P., Heliövaara, M., and Vartiainen, E. Associations of body mass index and obesity with physical activity, food choices, alcohol intake, and smoking in the 1982–1997 FINRISK Studies. *The American journal of clinical nutrition*, **2002**, *75*, 809–817.	0 portions/ week<br>1–3 portions/ week<br>4–9 portions/ week<br>≥10 portions/ week
Lukasiewicz, E., Mennen, L. I., Bertrais, S., Arnault, N., Preziosi, P., Galan, P., Hercberg, S. Alcohol intake in relation to body mass index and waist-to-hip ratio: the importance of type of alcoholic beverage. *Public health nutrition* **2005**, *8*, 315–320.	Abstainers<br>0-12 g/day<br>12-24 g/day<br>>42 g/day
Wannamethee, S. G., Shaper, A. G., Whincup, P. H. Alcohol and adiposity: effects of quantity and type of drink and time relation with meals. **International Journal of Obesity 2005**, *29*, 1436–1444.	None/<1 drinks/week<br>1–6 drinks /week<br>7–20 drinks /week<br>21–34 drinks /week<br>35+ drinks /week
Oh, S. S., Kim, W., Han, K. T., Park, E. C., Jang, S. I. Alcohol consumption frequency or alcohol intake per drinking session: Which has a larger impact on the metabolic syndrome and its components? *Alcohol* **2018**, *71*, 15–23.	Abstention<br>Less than once a month<br>Once a month<br>2-4 times per month<br>2-3 times per week<br>More than 4 times per week
Sayon-Orea, C., Bes-Rastrollo, M., Nuñez-Cordoba, J. M., Basterra-Gortari, F. J., Beunza, J. J., Martinez-Gonzalez, M. A. Type of alcoholic beverage and incidence of overweight/obesity in a Mediterranean cohort: the SUN project. *Nutrition* **2011**, *27*, 802–808.	Non-drinkers<br><1 drink/week<br>1 to <2 drinks/ week<br>2 to <7 drinks/ week<br>>7 drinks/ week
Tayie, F. A., Beck, G. L. Alcoholic beverage consumption contributes to caloric and moisture intakes and body weight status. *Nutrition* **2016**, *32*, 799–805.	Nondrinker<br>1 drink/d<br>2 drinks/d<br>3 drinks/d<br>>4 drinks/d
Ryu, M., Kimm, H., Jo, J., Lee, S. J., Jee, S. H. Association between alcohol intake and abdominal obesity among the Korean population. *Epidemiology and health* **2010**, *32*.	Non-drinkers<br>1–10 g of alcohol per day<br>11–20 g of alcohol per day<br>>20 g of alcohol per day
Sa, J., Russell, M., Weintruab, M. R., Seo, D. C., Chaput, J. P., Habib, M. Gender and Racial/Ethnic Differences in the Association Between Alcohol Drinking Patterns and Body Mass Index—the National Health and Nutrition Examination Survey, 1999–2010. *Journal of racial and ethnic health disparities* **2019**, *6*, 301–311.	Abstainers<br>1–2 drinks/day<br>3–4 drinks/day<br>≥ 5 drinks/day
Ruidavets, J. B., Bataille, V., Dallongeville, J., Simon, C., Bingham, A., Amouyel, P., ... & Ferrières, J. Alcohol intake and diet in France, the prominent role of lifestyle. *European heart journal* **2004**, *25*, 1153–1162.	Non-drinkers<br>1–19 g /day<br>20–39 g /day<br>40–59 g /day<br>>59 g /day

Table S2. Multivariable-adjusted associations between alcohol consumption and BMI (including underweight participants) and body fat percentage in the UK Biobank according to UK Drinking Guidelines categorization (*n* = 282 572)

Alcohol consumption	Model 1		Model 2	
	**Coefficient (95% CI)**	***P***	**Coefficient (95 %CI)**	***P***
BMI				
Never drinker	Referent	<0.001	Referent	<0.001
Previous drinker	−0.001 (−0.11, 0.11)		−0.09 (−0.20, 0.01)	
Within guidelines	−1.22 (−1.29, −1.14)		−1.06 (−1.13, −0.98)	
Hazardous	−0.73 (−0.81, -−0.64)		−0.63 (−0.71, −0.55)	
Harmful	0.08 (-0.07, 0.24)		−0.16 (−0.32, −0.01)	
Body fat percentage				
Never drinker	Referent	0.50	Referent	0.52
Previous drinker	0.15 (−0.06, 0.37)		0.15 (−0.06, 0.36)	
Within guidelines	0.06 (−0.09, 0.21)		0.06 (−0.09, 0.22)	
Hazardous	0.06 (−0.10, 0.22)		0.07 (−0.09, 0.23)	
Harmful	0.21 (−009, 0.52)		0.22 (−0.09, 0.52)	

Generalised linear model coefficient; mean differences (in risk factor values) between the reference category (never drinker) and each of the other alcohol consumption categories.

Model 1 is adjusted for age and sex only. Model 2 is adjusted for age, sex, smoking status, sleep (hrs/night), sedentary behaviour (hrs/day), illness (major cardiovascular disease or cancer), physical activity, Townsend deprivation index and daily fruit and vegetable consumption.

Alcohol consumption categories are based on the average weekly intake of standard drinks relative to UK guidelines. In the UK, one standard drink equals to 10mL of pure alcohol. Within guidelines: <14 units/week in women and <21 units/week in men; hazardous: 14-35 units/week in women and 21-49 units/week in men; harmful: >35 units/week in women and >49 units/week in men.

Body mass index (BMI) = Weight (kg)/height (m^2). A BMI ≥ 25 was considered overweight and ≥30 was considered obese.

Physical activity (PA) was classified based on the Metabolic Equivalent Task (MET) scores of participants' responses to the International Physical Activity Questionnaire (IPAQ) as inactive (≤7.5 MET-hour/week), active at the lower PA guideline (>7.5 MET-hour/week), or active at the upper PA guideline (>15 MET-hour/week). MET-hours/week were calculated based on the average number of minutes per day spent walking for any purpose, minutes/day in moderate PA and vigorous PA.

Townsend deprivation index scores were derived from national census data. Each participant was assigned a score relative to the output area in which their postcode was located. Higher scores reflects a higher degree of socioeconomic deprivation

Table S3. Multivariable-adjusted associations between alcohol consumption and BMI and body fat percentage in the UK Biobank with adjustment for education (*n* = 171 772)

	**Model 1**		**Model 2**	
	**Coefficient (95% CI)**	***P***	**Coefficient (95 %CI)**	***P***
**Alcohol consumption**				
BMI				
Never drinker	Referent	<0.001	Referent	<0.001
Previous drinker	0.06 (−0.07, 0.19)		−0.05 (−0.18, 0.08)	
Within guidelines	−1.24 (−1.34, −1.14)		−1.02 (−1.12, −0.93)	
Hazardous	−0.86 (−0.96, −0.76)		−0.66 (−0.77, −0.56)	
Harmful	−0.44 (−0.63, −0.24)		−0.50 (−0.69, −0.31)	
Body fat percentage				
Never drinker	Referent	0.59	Referent	0.58
Previous drinker	0.23 (−0.03, 0.49)		0.23 (−0.03, 0.49)	
Within guidelines	0.06 (−0.12, 0.25)		0.08 (−0.11, 0.27)	
Hazardous	0.08 (−0.12, 0.28)		0.09 (−0.11, 0.29)	
Harmful	0.31 (−0.07, 0.68)		0.32 (−0.06, 0.69)	

Generalised linear model coefficient; mean differences (in risk factor values) between the reference category (never drinker) and each of the other alcohol consumption categories.
Model 1 is adjusted for age and sex only. Model 2 is adjusted for age at completing education, smoking status, sleep (hrs/night), sedentary behaviour (hrs/day), illness (major cardiovascular disease or cancer), physical activity, Townsend deprivation index and daily fruit and vegetable consumption.
Alcohol consumption categories are based on the average weekly intake of standard drinks relative to UK guidelines. In the UK, one standard drink equals to 10mL of pure alcohol. Within guidelines: <14 units/week in women and <21 units/week in men; hazardous: 14-35 units/week in women and 21-49 units/week in men; harmful: >35 units/week in women and >49 units/week in men.
Body mass index (BMI) = Weight (kg)/height (m^2). A BMI ≥ 25 was considered overweight and ≥30 was considered obese.
Physical activity (PA) was classified based on the Metabolic Equivalent Task (MET) scores of participants' responses to the International Physical Activity Questionnaire (IPAQ) as inactive (≤7.5 MET-hour/week), active at the lower PA guideline (>7.5 MET-hour/week), or active at the upper PA guideline (>15 MET-hour/week). MET-hours/week were calculated based on the average number of minutes per day spent walking for any purpose, minutes/day in moderate PA and vigorous PA.
Townsend deprivation index scores were derived from national census data. Each participant was assigned a score relative to the output area in which their postcode was located. Higher scores reflects a higher degree of socioeconomic deprivation.

Table S4. Multiple logistic regression describing the associations between alcohol consumption and overweight including obesity in the UK Biobank (*n* = 281 588).

	Cases/*n*	Model 1		Model 2	
		**Odds ratio (95% CI)**	***P***	**Odds ratio (95 %CI)**	***P***
**Alcohol consumption**					
Overweight and obese					
Never drinker	9003/13 276	1.00 (referent)	<0.001	1.00 (referent)	<0.001
Previous drinker	8106/11 792	0.92 (0.87, 0.97)		0.85 (0.80, 0.89)	
Within guidelines	110 173/176 704	0.70 (0.68, 0.73)		0.70 (0.67, 0.73)	
Hazardous	54 688/75 866	0.90 (0.87, 0.94)		0.87 (0.83, 0.91)	
Harmful	2917/3950	1.10 (1.01, 1.19)		0.99 (0.91, 1.08)	
Obese					
Never drinker	3811/13 276	1.00 (referent)	<0.001	1.00 (referent)	<0.001
Previous drinker	3504/11 792	1.00 (0.95, 1.05)		0.92 (0.86, 0.97)	
Within guidelines	33 740/176 704	0.56 (0.54, 0.59)		0.59 (0.57, 0.61)	
Hazardous	17 965/75 866	0.69 (0.66, 0.72)		0.70 (0.67, 0.73)	
Harmful	1230/3950	1.05 (0.97, 1.13)		0.95 (0.88, 1.03)	

Cases; number of participants that are overweight and obese or obese, n; number of participants in sample.
Model 1 is adjusted for age and sex only. Model 2 is adjusted for smoking status, sleep (hrs/night), sedentary behaviour (hrs/day), illness (major cardiovascular disease or cancer), physical activity, Townsend deprivation index and daily fruit and vegetable consumption.
Alcohol consumption categories are based on the average weekly intake of standard drinks relative to UK guidelines. In the UK, one standard drink equals to 10mL of pure alcohol. Within guidelines: <14 units/week in women and <21 units/week in men; hazardous: 14–35 units/week in women and 21–49 units/week in men; harmful: >35 units/week in women and >49 units/week in men.
Body mass index (BMI) = Weight (kg)/height (m^2). A BMI ≥ 25 was considered overweight and ≥30 was considered obese.
Physical activity (PA) was classified based on the Metabolic Equivalent Task (MET) scores of participants' responses to the International Physical Activity Questionnaire (IPAQ) as inactive (≤7.5 MET-hour/week), active at the lower PA guideline (>7.5 MET-hour/week), or active at the upper PA guideline (>15 MET-hour/week). MET-hours/week were calculated based on the average number of minutes per day spent walking for any purpose, minutes/day in moderate PA and vigorous PA.
Townsend deprivation index scores were derived from national census data. Each participant was assigned a score relative to the output area in which their postcode was located. Higher scores reflects a higher degree of socioeconomic deprivation.

Table S5. Multivariable-adjusted associations between alcohol consumption and BMI and body fat percentage in the UK Biobank with combined never/previous drinkers (*n* = 280, 183).

	Model 1		Model 2	
	**Coefficient (95% CI)**	***P***	**Coefficient (95 %CI)**	***P***
**Alcohol consumption**				
BMI				
Never/previous drinker	Referent	0.05	Referent	0.13
Within guidelines	–1.33 (–1.39, –1.28)		–1.07 (–1.13, –1.01)	
Hazardous	–0.84 (–0.91, –0.78)		–0.65 (–0.71, –0.59)	
Harmful	–0.08 (–0.23, 0.06)		–0.23 (–0.37, –0.08)	
Body fat percentage				
Never/previous drinker	Referent	0.80	Referent	0.74
Within guidelines	–0.01 (–0.13, 0.10)		–0.003 (–0.12, 0.11)	
Hazardous	–0.01 (–0.14, 0.11)		–0.004 (–0.13, 0.12)	
Harmful	0.14 (–0.15, 0.43)		0.15 (–0.14, 0.44)	

Generalised linear model coefficient; mean differences (in risk factor values) between the reference category (never drinker) and each of the other alcohol consumption categories.
Model 1 is adjusted for age and sex only. Model 2 is adjusted for smoking status, sleep (hrs/night), sedentary behaviour (hrs/day), illness (major cardiovascular disease or cancer), physical activity, Townsend deprivation index and daily fruit and vegetable consumption.
Alcohol consumption categories are based on the average weekly intake of standard drinks relative to UK guidelines. In the UK, one standard drink equals to 10mL of pure alcohol. Within guidelines: <14 units/week in women and <21 units/week in men; hazardous: 14–35 units/week in women and 21–49 units/week in men; harmful: >35 units/week in women and >49 units/week in men.

Body mass index (BMI) = Weight (kg)/height (m^2). A BMI ≥ 25 was considered overweight and ≥30 was considered obese.
Physical activity (PA) was classified based on the Metabolic Equivalent Task (MET) scores of participants' responses to the International Physical Activity Questionnaire (IPAQ) as inactive (≤7.5 MET-hour/week), active at the lower PA guideline (>7.5 MET-hour/week), or active at the upper PA guideline (>15 MET-hour/week). MET-hours/week were calculated based on the average number of minutes per day spent walking for any purpose, minutes/day in moderate PA and vigorous PA.
Townsend deprivation index scores were derived from national census data. Each participant was assigned a score relative to the output area in which their postcode was located. Higher scores reflects a higher degree of socioeconomic deprivation.

Table S6. Multivariable-adjusted associations between types of alcoholic beverages consumed and BMI (including underweight participants) and body fat percentage in the UK Biobank (*n* = 282 572).

	**Model 1**		**Model 2**	
	**Coefficient (95% CI)**	***P***	**Coefficient (95% CI)**	***P***
**Alcohol consumption** (ref: not drinking this drink type)				
BMI				
Red wine	−0.71 (−0.75, −0.68)	<0.001	−0.52 (−0.55, −0.48)	<0.001
Champagne/white wine	−0.46 (−0.49, −0.43)	<0.001	−0.37 (−0.41, −0.34)	<0.001
Beer/cider	0.20 (0.17, 0.24)	<0.001	0.21 (0.17, 0.25)	<0.001
Spirits	0.66 (0.63, 0.70)	<0.001	0.56 (0.52, 0.59)	<0.001
Fortified wine	−0.24 (−0.30, −0.19)	<0.001	−0.18 (−0.24, −0.13)	<0.001
Other alcohol	0.58 (0.33, 0.83)	<0.001	0.30 (0.06, 0.55)	0.01
Body fat percentage				
Red wine	0.03 (−0.03, 0.10)	0.34	0.03 (0.04, 0.10)	0.40
Champagne/white wine	−0.06 (−0.12, 0.01)	0.07	−0.07 (−0.13, 0.00)	0.05
Beer/cider	−0.03 (−0.10, 0.05)	0.50	−0.04 (−0.12, 0.04)	0.33
Spirits	0.01 (−0.06, 0.08)	0.74	0.01 (−0.06, 0.08)	0.79
Fortified wine	0.01 (−0.10, 0.12)	0.83	0.02 (−0.09, 0.13)	0.77
Other alcohol	0.21 (−0.28, 0.70)	0.40	0.23 (0.26, 0.72)	0.35

Generalised linear model coefficient; mean differences (in risk factor values) between participants who did not consume the relevant alcohol type (the referent) and participants who reported consuming the relevant alcohol type.

Model 1 is adjusted for age and sex only. Model 2 is adjusted for age, sex, overall alcohol consumption, smoking status, sleep (hrs/night), sedentary behaviour (hrs/day), illness (major cardiovascular disease or cancer), physical activity, Townsend deprivation index and daily fruit and vegetable consumption.

Overall alcohol consumption categories are based on the average weekly intake of standard drinks relative to UK guidelines. In the UK, one standard drink equals to 10mL of pure alcohol. Within guidelines: <14 units/week in women and <21 units/week in men; hazardous: 14-35 units/week in women and 21-49 units/week in men; harmful: >35 units/week in women and >49 units/week in men.

Body mass index (BMI) = Weight (kg)/height (m^2). A BMI ≥ 25 was considered overweight and ≥30 was considered obese.

Physical activity (PA) was classified based on the Metabolic Equivalent Task (MET) scores of participants' responses to the International Physical Activity Questionnaire (IPAQ) as inactive (≤7.5 MET-hour/week), active at the lower PA guideline (>7.5 MET-hour/week), or active at the upper PA guideline (>15 MET-hour/week). MET-hours/week were calculated based on the average number of minutes per day spent walking for any purpose, minutes/day in moderate PA and vigorous PA.

Townsend deprivation index scores were derived from national census data. Each participant was assigned a score relative to the output area in which their postcode was located. Higher scores reflects a higher degree of socioeconomic deprivation.

Table S7. Multiple logistic regression describing the associations between types of alcoholic beverages and overweight and obesity in the UK Biobank (*n* = 281, 588).

	Model 1		Model 2	
	**Odds ratio (95% CI)**	***P***	**Odds ratio (95 %CI)**	***P***
**Alcohol consumption**				
Overweight and obese				
Red wine	0.81 (0.80, 0.82)	<0.001	0.87 (0.86, 0.89)	<0.001
Champagne/white wine	0.87 (0.85, 0.88)	<0.001	0.91 (0.89, 0.92)	<0.001
Beer/cider	1.14 (1.12, 1.16)	<0.001	1.17 (1.14, 1.19)	<0.001
Spirits	1.34 (1.31, 1.36)	<0.001	1.28 (1.26, 1.30)	<0.001
Fortified wine	0.89 (0.87, 0.92)	<0.001	0.94 (0.91, 0.96)	<0.001
Other alcohol	1.16 (1.02, 1.31)	0.02	1.08 (0.95, 1.23)	0.22
Obese				
Red wine	0.70 (0.68, 0.71)	<0.001	0.79 (0.77, 0.80)	<0.001
Champagne/white wine	0.80 (0.78, 0.81)	<0.001	0.86 (0.85, 0.88)	<0.001
Beer/cider	1.06 (1.04, 1.08)	<0.001	1.15 (1.13, 1.18)	<0.001
Spirits	1.30 (1.28, 1.33)	<0.001	1.27 (1.25, 1.30)	<0.001
Fortified wine	0.88 (0.85, 0.91)	<0.001	0.92 (0.89, 0.95)	<0.001
Other alcohol	1.32 (1.16, 1.50)	<0.001	1.22 (1.07, 1.40)	0.003

Model 1 is adjusted for age and sex only. Model 2 is adjusted for overall alcohol consumption, smoking status, sleep (hrs/night), sedentary behaviour (hrs/day), illness (major cardiovascular disease or cancer), physical activity, Townsend deprivation index and daily fruit and vegetable consumption.
Alcohol consumption categories are based on the average weekly intake of standard drinks relative to UK guidelines. In the UK, one standard drink equals to 10mL of pure alcohol. Within guidelines: <14 units/week in women and <21 units/week in men; hazardous: 14-35 units/week in women and 21–49 units/week in men; harmful: >35 units/week in women and >49 units/week in men.
Body mass index (BMI) = Weight (kg)/height (m^2). A BMI ≥ 25 was considered overweight and ≥30 was considered obese.
Physical activity (PA) was classified based on the Metabolic Equivalent Task (MET) scores of participants' responses to the International Physical Activity Questionnaire (IPAQ) as inactive (≤7.5 MET-hour/week), active at the lower PA guideline (>7.5 MET-hour/week), or active at the upper PA guideline (>15 MET-hour/week). MET-hours/week were calculated based on the average number of minutes per day spent walking for any purpose, minutes/day in moderate PA and vigorous PA.
Townsend deprivation index scores were derived from national census data. Each participant was assigned a score relative to the output area in which their postcode was located. Higher scores reflect a higher degree of socioeconomic deprivation.
